# The Utilization of Artificial Burrows by Plateau Pikas Under Rapid Vegetation Restoration

**DOI:** 10.1002/ece3.72526

**Published:** 2025-11-16

**Authors:** Rui Zhang, Wei Liu

**Affiliations:** ^1^ College of Coastal Agricultural Sciences Guangdong Ocean University Zhanjiang China; ^2^ Northwest Institute of Plateau Biology Chinese Academy of Sciences Xining China

**Keywords:** alpine meadow, artificial burrow, *Ochotona curzoniae*, Qinghai‐Tibet plateau, spatial adaptation strategy

## Abstract

Vegetation changes often elicit adaptive responses in herbivores. However, how burrow‐dwelling animals utilize the potential underground spatial resource in response to rapid aboveground vegetation changes remains poorly understood. In 2016, we conducted field experiments on a degraded alpine meadow on the Qinghai‐Tibet Plateau using plateau pikas ( *Ochotona curzoniae* ) as model species. The research area was 1.5 ha in total, of which half received fertilization to simulate vegetation restoration. We constructed 288 artificial burrows of four types (length: 10 cm/20 cm; inclination: 45°/90°) at the beginning of the growing season and monitored the utilization throughout the season. The results showed that artificial burrow utilization rate in the form of latrine pits (shallow pits used for defecation) was higher than that of shallow duck holes (shallow refuge holes used for emergency predator evasion) and active burrows. Artificial burrow use did not differ significantly between restored and control areas during the growing season. The results indicate that plateau pikas may not prefer to cope with rapid vegetation changes by adjusting the use of spatial resources. Instead, burrow accessibility and scent‐marking potential—driven by morphological preferences—are the primary determinants shaping their spatial resource selection. Our findings enhance understanding of the spatial adaptation of burrow‐dwelling animals to rapid vegetation changes and provide insights for their conservation.

## Graphical abstract

Vegetation changes often elicit adaptive responses in herbivores. Plateau pikas do not prefer to cope with rapid vegetation changes by adjusting the use of spatial resources. Instead, burrow accessibility and scent‐marking potential—driven by morphological preferences—are the primary determinants shaping their spatial resource selection.

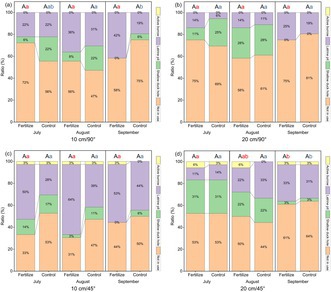

## 1 Introduction

The vegetation on the Qinghai‐Tibet Plateau is undergoing extensive changes, mainly manifested as grassland degradation (Gao et al. 2016; Xu and Liu 2007). Simultaneously, human interventions such as vegetation restoration and grazing prohibition to prevent degradation, have also altered the grassland vegetation (Cai et al. 2015; Zhou et al. 2024). Vegetation restoration is a standard approach for rapidly enhancing ecological functions in degraded ecosystems (Li et al. 2011; Xu et al. 2023). Changes in vegetation inevitably exert profound impacts on wildlife. Previous studies have primarily focused on animal responses to different plant communities (Xu et al. 2024) or vegetation degradation (Wang et al. 2024; Wu et al. 2015). Limited attention has been given to how animals adapt to vegetation restoration, especially during the rapid restoration processes driven by human intervention.

Grassland ecosystems are frequently shaped by a key functional group of social, burrowing, herbivorous mammals (Davidson et al. 2012). In alpine meadow ecosystems, the plateau pika ( *Ochotona curzoniae* ) serves as a keystone species and ecosystem engineer (Lai and Smith 2003; Smith and Foggin 1999). A moderate density of plateau pika populations is essential for maintaining biodiversity and soil health in alpine meadows (Cui et al. 2024). Plateau pikas are not only of great ecological importance in alpine meadow ecosystems, but also exhibit significant research value due to their adaptability and survival strategies in response to environmental changes. Additionally, vegetation management aimed at controlling plateau pika density has been frequently proposed as a strategy to mitigate alpine meadow degradation (Li et al. 2023; Song et al. 2023; Wei et al. 2023). However, previous studies have predominantly focused on the effects of plateau pikas on meadow vegetation (Chen et al. 2025; Qin et al. 2021; Yao et al. 2022), while research on their responses to vegetation management remains limited. Plateau pikas live in cohesive and highly socially integrated family groups that occupy an elaborate, interconnected burrow system. The restricted scope of activities makes the plateau pika an excellent species for studying animal responses to rapid vegetation changes.

Research on plateau pika responses to vegetation changes has primarily focused on the effects of vegetation on their diet composition and behavioral patterns. Changes in vegetation composition directly influence the diet of herbivores, and adjusting food resources is a critical adaptation strategy for herbivores (Dong et al. 2025). In response to alpine meadow vegetation degradation, plateau pikas modify their diet to increase protein intake (Wang et al. 2024; Wu et al. 2023). Nonetheless, most species exhibit stronger responses to vegetation structure than to composition (George and Zack 2001). Degraded alpine meadow vegetation, characterized by reduced height, reduced the warning frequency of plateau pika (Li et al. 2023). The degradation also results in the expansion of plateau pika territory core areas and increased digging behavior (Wang et al. 2023).

Regulation of spatial resources is another important strategy for animals to cope with vegetation changes. Human‐induced vegetation landscape transformations can profoundly change rodent burrow abundance and distribution (Surkova et al. 2019). Adjusting the design and distribution of burrow systems to optimize spatial resource utilization is also a key component of the plateau pika survival strategies (Zhang and Liu 2024a; Zhang, Xu, and Liu 2018). The structure of their burrow systems often varies with field types (Qin et al. 2018). Changes in vegetation have also prompted plateau pikas to adjust their utilization of spatial resources. For example, plateau pikas prefer robust burrow entrances and may adjust their burrow density in response to the coverage of different plant functional groups (Zhang and Liu 2024b). The aforementioned studies are based on slow vegetation changes under natural conditions; little research exists on plateau pika responses to rapid vegetation changes in potential spatial resource utilization, particularly for artificial burrow resources.

Restoring animal populations through the creation of artificial burrows is a common practice (Souter et al. 2004). This approach primarily targets endangered species or populations that are too small to fulfill their ecological roles (McCullough Hennessy et al. 2016). Using ecosystem engineers to restore ecological systems is a significant approach (Byers et al. 2006). Although most alpine meadow ecosystems currently exhibit an overabundant density of plateau pikas, there are areas where ecosystem imbalances, caused by excessive culling as scapegoats, necessitate the restoration of pika populations (Delibes‐Mateos et al. 2011; Smith et al. 2019). However, large‐scale burrow collapse impeded the potential recolonization of these areas. Understanding the utilization of artificial burrows by plateau pikas is crucial for the conservation of alpine meadow ecosystems in these areas.

In this study, we investigated the differences in artificial burrow utilization by plateau pikas under different vegetation growth rates and aimed to explore their spatial response strategies to rapid vegetation restoration. We hypothesized that enhanced vegetation increases predation risk for plateau pikas and intensifies their competition for burrow resources. Considering that modifying artificial burrows requires large amounts of energy, the competition may manifest more as the occupation of burrow resources through marking. This study contributes to the understanding of the adaptation mechanisms of plateau pikas to vegetation management, and provides insights for the conservation of burrowing animals.

## 2 Materials and Methods

### 2.1 Site Description

The experimental site was situated in a *Kobresia pygmaea* meadow in the northeastern Qinghai‐Tibet Plateau in Qilian County, Qinghai Province, China (100°14′3.43′′ E, 37°58′51.84′′; altitude 3660 m). The annual average temperature was −3°C, with an average precipitation of 420 mm, approximately 2500 h of sunshine annually, and no absolute frost‐free period throughout the year. The sample plot was established in a winter pasture and enclosed with iron wire to prevent livestock disturbance. According to the degradation index developed by Gao et al. (2010), the study area was lightly degraded. Two years prior to the study (2014), the plateau pika population was exterminated in this area during winter. At the end of the breeding season in 2015, the plateau pika population density was approximately 40/hm^2^, and the natural active burrow density was approximately 1600/hm^2^.

### 2.2 Experimental Design

**FIGURE 1:**
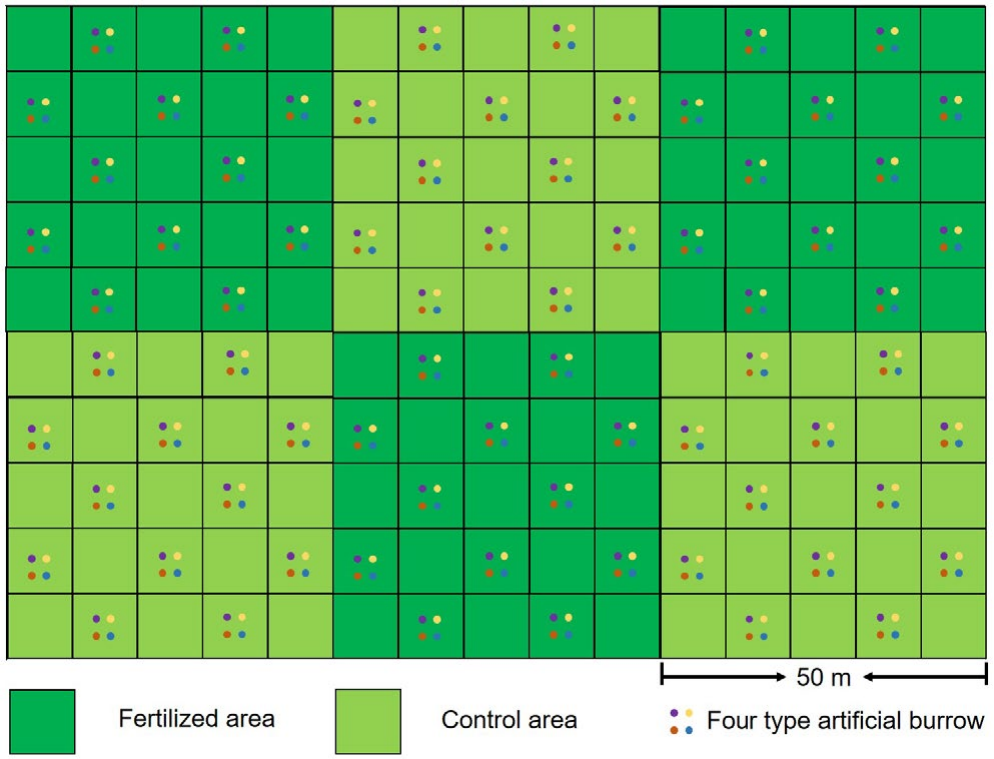
Schematic diagram of the plots and artificial burrows layout.

A 100 m × 150 m plot was selected from a meadow characterized by flat terrain and uniform vegetation. The plot was divided into six 50 m × 50 m square subplots, with half of them fertilized to alter vegetation growth (Figure 1). A plateau pika family typically occupies a home range of approximately 300 m^2^ (Qu et al. 2007), implying that 8–9 plateau pika families inhabit each subplot. The subplot was further divided into 10 m × 10 m quadrats, with thin iron wires marking the boundaries, and artificial burrows were drilled in 12 of these quadrats spaced apart. A total of 288 artificial burrows were drilled into bare soil patches within the plots. Four artificial burrows (one of each type) were established in each quadrat, spaced 1.0–1.5 m apart and positioned more than 1 m from existing natural burrow entrances.

**FIGURE 2:**
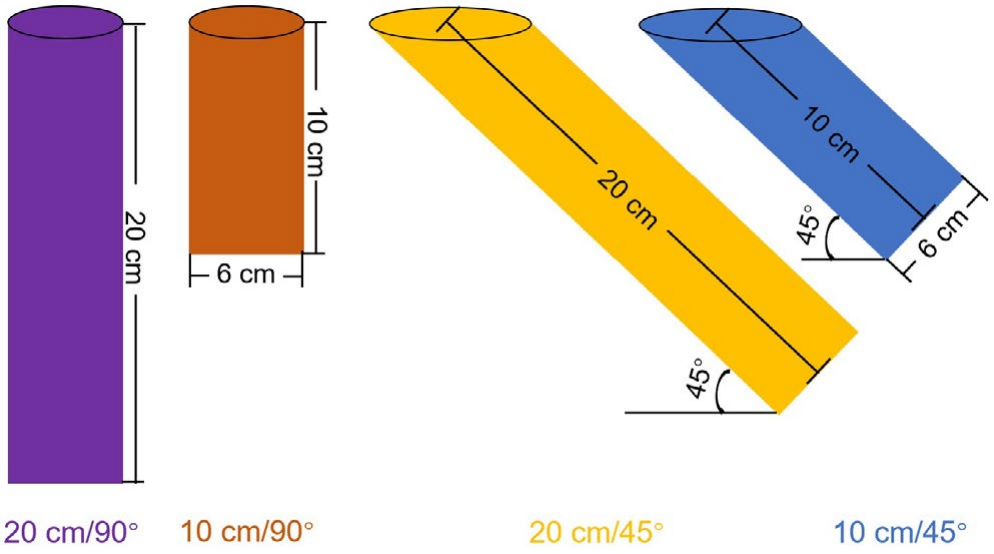
Schematic diagram of the four types of artificial burrows.

Four types of burrows were designed using a two‐factor randomized block design, varying in inclination (45° and 90°) and length (10 and 20 cm), to examine the effects of burrow morphology on artificial burrow utilization (Figure 2). Under natural conditions, sunny‐facing burrow entrances are the most common, with most having inclinations of less than 55° (Wei et al. 2013). Additionally, 45° inclinations are commonly used for artificial burrows in other small burrow‐dwelling animals (McCullough Hennessy et al. 2016). Therefore, all inclined burrow entrances were oriented southward, with inclinations of 45°. The body width of the plateau pika is approximately 5.4 cm (Wei and Zhang 2018), and the burrow tunnel diameter was approximately 6–7 cm (Li et al. 2025; Qin et al. 2018); therefore, a soil drill with an internal diameter of 5 cm was used to construct the artificial burrows. The diameter of the artificial burrows, influenced by the pressure from the soil drill, ranges from 5.5 to 6 cm.

### 2.3 Artificial Burrow Use Form Classification and Judgment

Artificial burrows were constructed in late April 2016. The use of the artificial burrows was evaluated on July 15, August 17, and September 7 in 2016. The utilization forms were categorized into three types: active burrows, latrine pits (shallow pits used for defecation, hereafter referred to as latrine pits), and shallow duck holes (shallow refuge holes used for emergency predator evasion, hereafter referred to as shallow duck holes). A burrow was classified as active if the total tunnel length increased by more than 30 cm, and the burrow bottom was not directly visible (sufficient to conceal an individual shorter than 20 cm). The presence of accumulated fresh fecal particles at the burrow bottom was the sole criterion for classifying it as a latrine pit. A burrow was classified as a shallow duck hole if there were fresh visible digging traces at the entrance, and the burrow bottom was directly visible without fecal accumulation. The classification priority was active burrows > latrine pits > shallow duck holes.

### 2.4 Vegetation Growth Regulation and Investigation

The subplots were fertilized twice with urea at a dosage of 5 g/m^2^ in early June and July of 2016 respectively to induce differences in vegetation growth. Vegetation was investigated in mid‐August using quincunx sampling, with a quadrat size of 0.5 m × 0.5 m. Vegetation coverage was measured using the point‐grid method with a 25‐grid layout. Community height was calculated as the weighted average height of four functional groups (grasses, sedges, weeds, and legumes) based on their coverage. The height of each functional group was determined as the mean height of five randomly selected plants (Zhang, Liu, and Xu 2018). The aboveground biomass of the entire 0.5 m × 0.5 m quadrat was measured by harvesting the aboveground plant components. The samples were oven‐dried at 65°C for 48 h and weighed.

### 2.5 Plateau Pika and Burrow Density Monitoring

Plateau pika and natural burrow numbers in the plots were monitored twice and once per month respectively from April to September in 2016. Plateau pika numbers were counted using telescopic observations during peak activity periods. The active state of burrows was assessed through direct observation. For detailed monitoring methods, please refer to our previous study (Zhang and Liu 2024a).

### 2.6 Statistical Analysis

Differences in vegetation conditions, population density, and burrow density between treatments were analyzed using the Mann–Whitney *U* test. A chi‐squared test was used to compare utilization differences between different treatments and artificial burrow types. When a burrow use form did not appear in a given treatment (i.e., observed count = 0), we merged that form with another within the same treatment that was ecologically or functionally similar. Merging was performed within treatment only, preserved the original levels' interpretability, and ensured that (1) each treatment retained at least two burrow use categories, and (2) all expected cell counts were at least five to meet the test's assumptions. If merging alone could not resolve sparse tables, we applied Fisher's exact test for 2 × 2 comparisons or exact/permutation alternatives. The significance threshold was set at *p* < 0.05, and all statistical analyses were conducted using SPSS software (version 23.0; SPSS Inc., Chicago, IL, USA).

## 3 Results

### 3.1 The Impact of Fertilization on Vegetation

**FIGURE 3:**
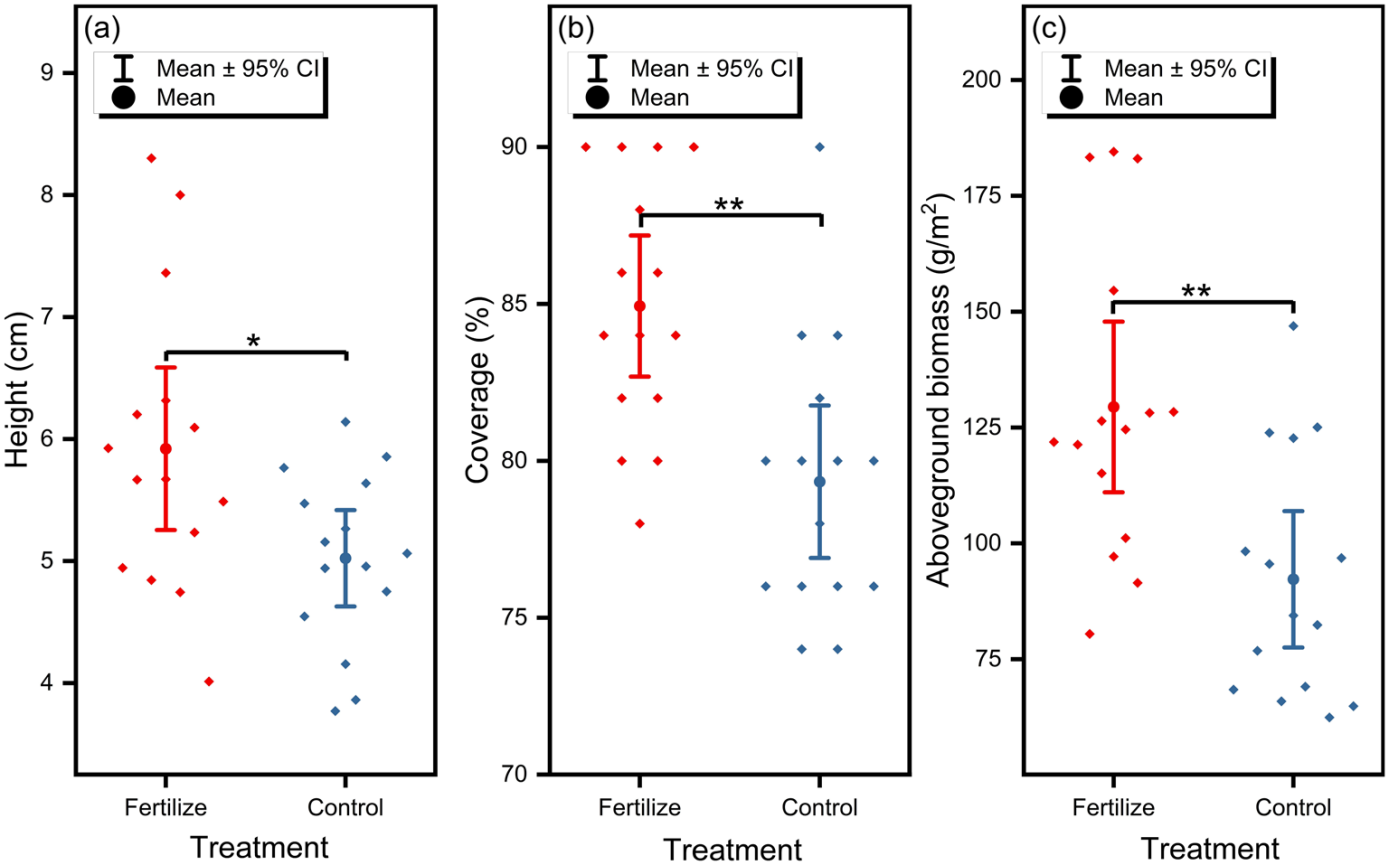
Vegetation (a) height, (b) coverage, and (c) aboveground biomass in fertilized and control plots. * and ** indicate significant (*p* < 0.05) and highly significant (*p* < 0.01) differences, respectively.

The fertilization had a significant impact on the growth of plants. Compared to the control area, the vegetation height, coverage, and aboveground biomass in the fertilization area were higher (Figure 3). The average vegetation height in the fertilized area was 5.9 cm, significantly higher than the 5.0 cm in the control area (Mann–Whitney *U* = 38.5, *n* = 15; *p* < 0.05); the average coverage in the fertilized area was 85%, significantly higher than the 79% in the control area (Mann–Whitney *U* = 63, *n* = 15; *p* < 0.01); the average aboveground biomass in the fertilized area was 129.6 g/m^2^, significantly higher than the 92.2 g/m^2^ in the control area (Mann–Whitney *U* = 42, *n* = 15; *p* < 0.01), representing increases of 18%, 7%, and 40%, respectively.

### 3.2 Population Density and Natural Burrow Resource Dynamics

**FIGURE 4:**
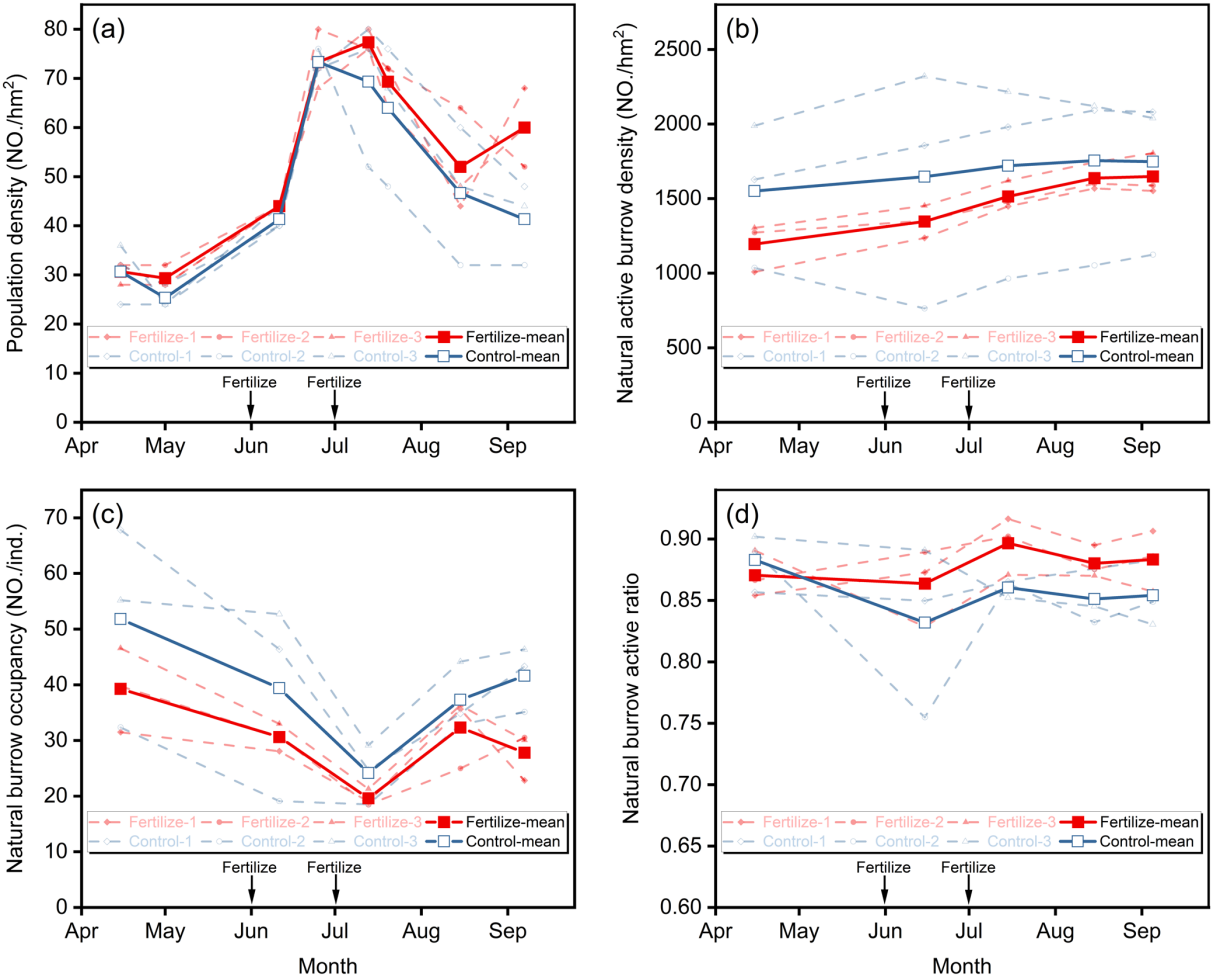
Dynamics in (a) population density, (b) natural active burrow density, (c) natural active burrow occupancy per individual, and (d) natural burrow active ratio.

Throughout the growing season, there were no statistically significant differences between the control and fertilized areas in population density (Figure 4a), natural active burrow density (Figure 4b), natural active burrow occupancy per individual (Figure 4c), and burrow active ratio (Figure 4d). Nonetheless, on average, natural active burrow density and natural burrow occupancy per individual were slightly lower in the fertilized area than in the control area, while population density exhibited the opposite trend—being slightly higher in the fertilized area.

### 3.3 Vegetation Effects on Artificial Burrows Utilization Pattern

**FIGURE 5:**
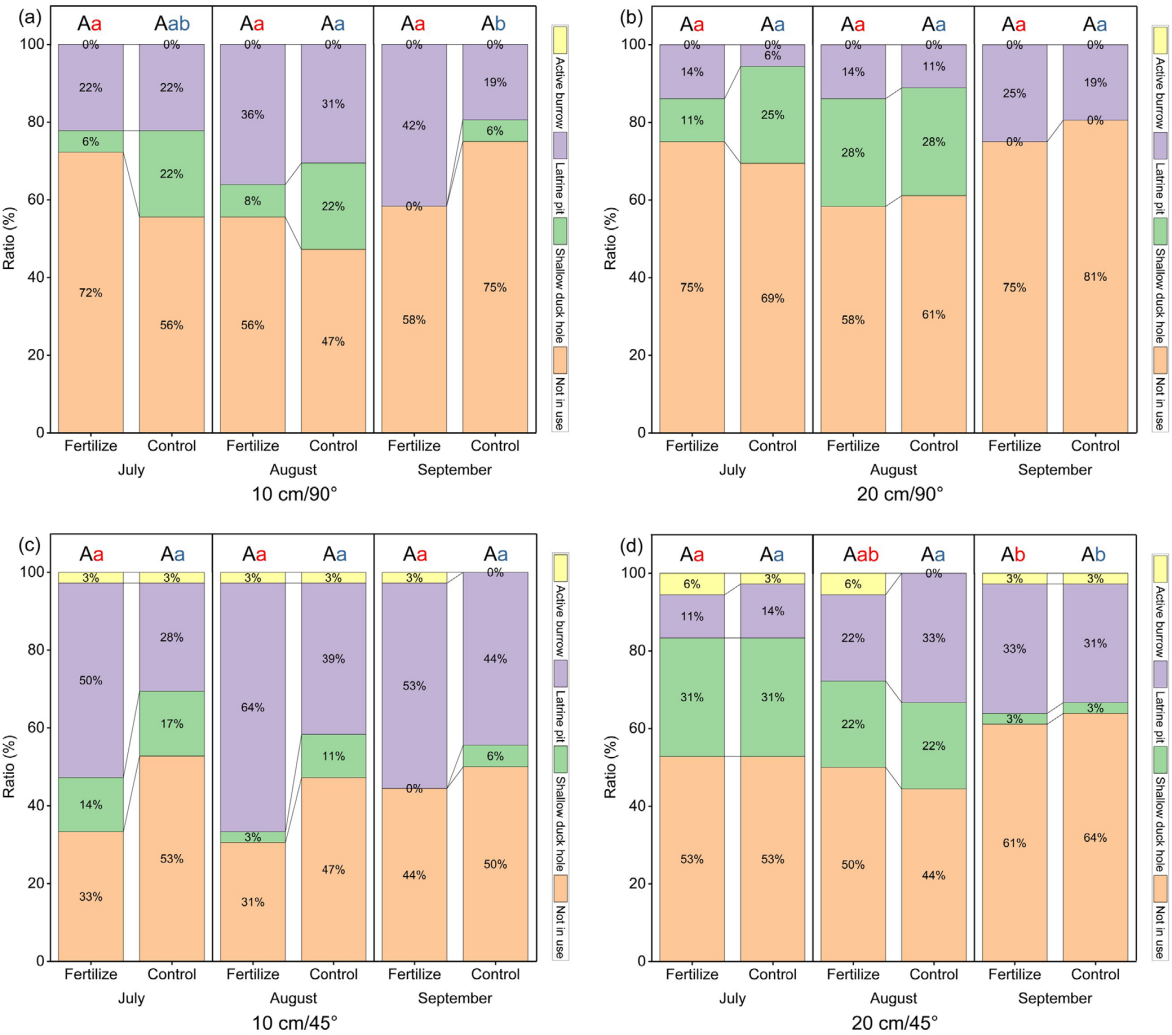
Effects of vegetation treatment on the utilization of four artificial burrow types [(a) 10 cm/90°; (b) 20 cm/90°; (c) 10 cm/45°; (d) 20 cm/45°]. Different letters denote significant differences: Uppercase letters indicate differences in utilization rates among burrow types within the same time period, while lowercase letters indicate differences in utilization rates for the same burrow type across time periods.

No significant differences in artificial burrow utilization were detected between control and fertilized areas for any burrow type throughout the growing season (Figure 5). The relative magnitude relationship of utilization rates in any forms between fertilized and control remained consistent over time. The artificial burrow utilization pattern remained relatively stable from July to August. However, a marked shift occurred in September (the end of the growing season), when utilization of shallow duck holes changed abruptly. During this period, utilization patterns of 10 cm/90° and 20 cm/45° burrows in control areas changed significantly. Meanwhile, the utilization rate in the form of shallow duck holes dropped to zero for 10 cm/90° and 10 cm/45° burrows in fertilized areas, as well as for 20 cm/90° burrows.

### 3.4 Burrow Morphology Effects on Artificial Burrows Utilization Pattern

**FIGURE 6:**
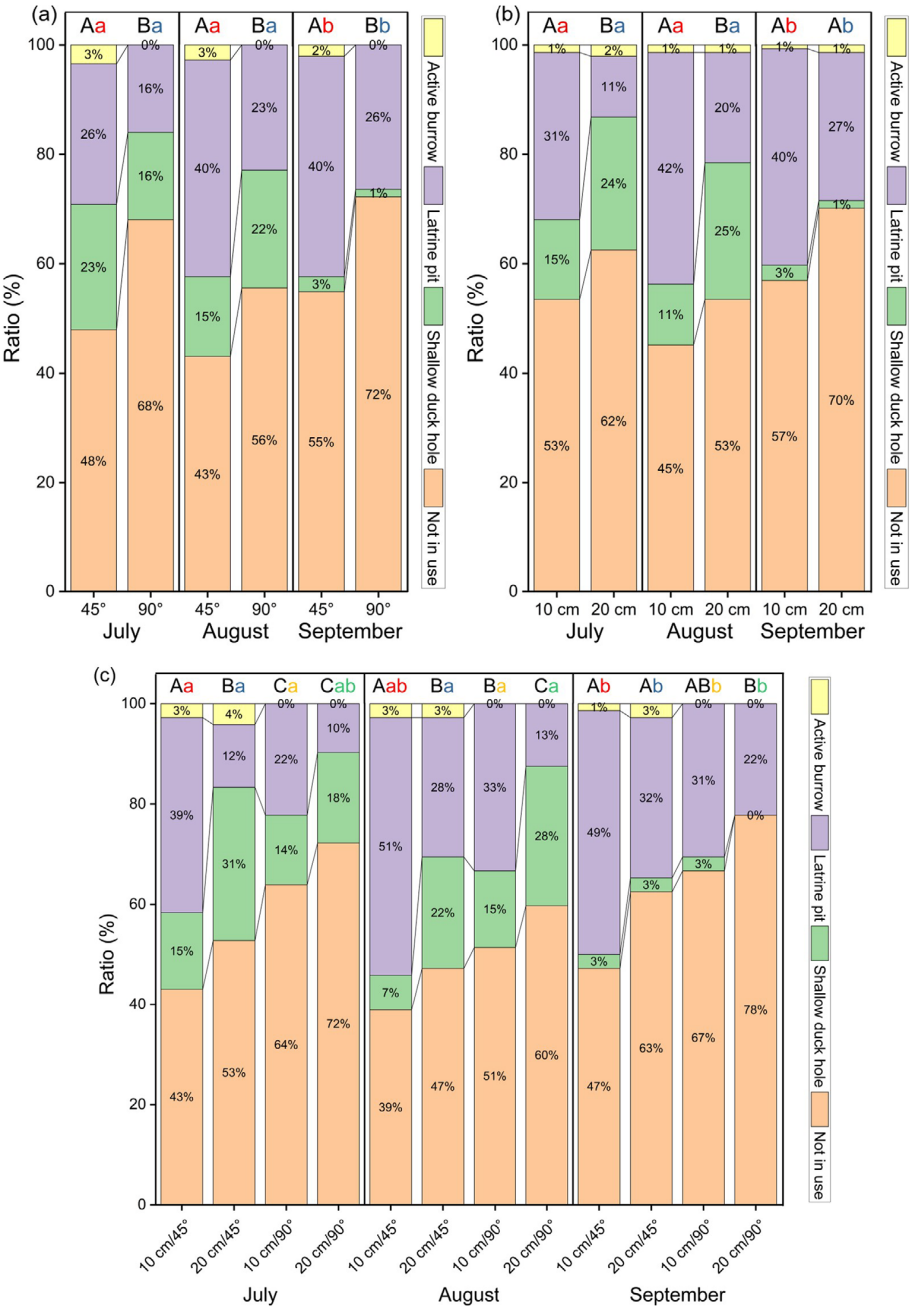
Effects of burrow morphology [(a) inclination; (b) length; (c) inclination and length] on the utilization of artificial burrows. Different letters denote significant differences: Uppercase letters indicate differences in utilization rates among burrow types within the same time period, while lowercase letters indicate differences in utilization rates for the same burrow type across time periods.

Both inclination and length had a significant influence on the utilization of artificial burrows. Burrows with a 45° inclination exhibited higher utilization rates than those with a 90° inclination across all three use forms; especially, all the artificial burrows utilized as active burrows were inclined (Figure 6a). On average, 20 cm‐long artificial burrows had higher utilization rates as shallow duck holes, but lower utilization as latrine pits compared to 10 cm‐long burrows (Figure 6b). Overall, the utilization preferences for the four types of artificial burrows ranked as follows: 10 cm/45° > 20 cm/45° > 10 cm/90° > 20 cm/90° (Figure 6c).

## 4 Discussion

### 4.1 The Utilization of Artificial Burrows by Plateau Pikas

The utilization rate of artificial burrows serves as a direct indicator of the effectiveness of increasing spatial resources. In our study, a large portion of the artificial burrows was not in use during the growing season. In contrast, burrowing Owls ( *Athene cunicularia* ) utilize artificial burrows at rates ranging from 83% to 96% (Menzel 2018). Several factors may explain the low utilization ratio of artificial burrows in our study. First, the high density of artificial burrows, accounting for 8%–25% of the active burrows in the plots, dilutes their utilization by plateau pikas. Second, the plateau pika population in our study area has already been established for a year. Intense digging activities typically occur when burrow‐dwelling animals relocate to new territories (Ivanter 2023). Consequently, there was limited urgency in utilizing artificial burrows. Third, the monitoring period for artificial burrows was limited to a single breeding season, providing insufficient time for plateau pikas to utilize them. Fourth, the artificial burrows were constructed on bare soil patches, which are not preferred by plateau pikas for burrow entrances (Zhang and Liu 2024b).

Artificial burrows exhibited utilization rates in the order of latrine pits > shallow duck holes > active burrows, consistent with our hypothesis. This utilization pattern markedly contrasts with natural conditions in the same region, which typically shows a 10:1:1 ratio of active burrows to latrine pits to shallow duck holes (Zhang and Liu 2024a). Plateau pikas' utilization of artificial burrows reflects an energy‐optimizing tradeoff between ecological benefits and energy costs. Latrine pits fulfill a critical and unique function: territorial marking (Smith et al. 1986; Wang and Dai 1990). Daily defecation incurs no additional metabolic costs while simultaneously providing territorial marking benefits. Shallow duck holes were utilized only for emergencies when plateau pikas cannot access their burrows in time (Zhang and Liu 2024a). When employed as shallow duck holes, it also requires a little modification to enhance artificial burrow accessibility. Functionally, shallow duck holes primarily reduce exposure risk; only active burrows could provide safe escape spaces. However, when employed as active burrows, much more energy is needed to excavate burrows.

The utilization ratio of artificial burrows as latrine pits and shallow duck holes was slightly lower than 1:1 in July and August. However, by September, toward the end of the growing season, the ratio for shallow duck holes declined significantly. This phenomenon suggests that, in the face of the upcoming winter, any non‐critical behavior that consumes much energy will be largely reduced.

### 4.2 Why Has Rapid Vegetation Restoration Not Triggered Spatial Utilization Adjustment?

The lack of significant differences in artificial burrow utilization patterns by plateau pikas despite substantial vegetation differences between treatments warrants deeper exploration of underlying mechanisms. Three potential mechanisms may explain this observation:

- First, the vegetation differences induced by fertilization, though statistically significant, may not be ecologically substantial enough to strongly influence burrowing behavior. Most animals exhibit significant responses to vegetation structure changes (George and Zack 2001). Research on the reintroduction of California ground squirrels ( *Otospermophilus beecheyi* ) demonstrated that mowing promotes intensive digging activities (McCullough Hennessy et al. 2016). Yellow‐bellied marmots ( *Marmota flaviventris* ) often select home burrows on open grassy or herb‐covered slopes (Svendsen 1976). Plateau pikas prefer to inhabit open spaces with lower vegetation heights (Tang et al. 2024), as taller vegetation obstructs visibility and elevates predation risk (Wei et al. 2023). Increased plant height can trigger behavioral adaptations such as extended vigilance periods, reduced alert distances, and accelerated movement speeds (Wei et al. 2022). However, we do not know to what extent vegetation changes can trigger these behavioral variations. The insufficient vegetation changes caused by fertilization may be the reason for the insignificant differences in their utilization of artificial burrows. Additionally, we only measured three aboveground vegetation parameters of height, coverage, and aboveground biomass. However, other specific vegetation structural factors might also influence plateau pika utilization of artificial burrows. It is possible that the differences in these factors between the two treatments were not significant. These warrant further investigation.
- Second, plateau pikas may prefer to cope with rapid vegetation changes by regulating aboveground behavior rather than utilizing underground spatial resources. Compared to the abovementioned behavioral adjustments, modifying subterranean spatial resources requires greater temporal and energetic investments. For relatively stable populations with established territories, the urgency for spatial resource reorganization is diminished. Such adjustments become more pronounced only when burrow resource requirements reach critical levels. It is evident that such a situation does not exist under current population density. However, if the population density of plateau pikas increases, their demand for burrow resources will become more urgent. Whether this would affect the results is unclear, and remains to be further investigated.
- Third, a temporal lag may exist in the response of plateau pikas to vegetation changes. Vegetation manipulation via fertilization often exhibits gradual effects on plant growth, and pika spatial adjustments are energetically and time‐costly. After the establishment of artificial burrows, there may be a considerable period during which vegetation differences between the two areas remain minimal. If given sufficient time, the importance of spatial resource adjustments in pika responses to vegetation changes may become more apparent. For example, previous research indicates that alpine meadow degradation intensifies the burrowing behavior of plateau pikas, resulting in increased burrow density (Wang et al. 2023). Long‐term monitoring is required to evaluate the role of underground spatial resource adjustment in responding to vegetation changes in future studies.

### 4.3 Plateau Pikas' Morphology Preference for Artificial Burrow

Overall, short and inclined artificial burrows were more attractive to plateau pikas than long and vertical burrows. The effect on scent marking, concealment, and energy saving may dominate the artificial burrow length selection of plateau pikas. Plateau pikas often use feces to mark their territories (Smith et al. 1986; Wang and Dai 1990). When feces accumulate in shallow artificial burrows, the scent disperses more effectively, enhancing territorial marking. Under natural conditions, the latrine pits of plateau pikas are typically shallow (Zhang and Liu 2024a). This preference for short artificial burrows as latrine pits aligns with their natural behavior. When functioning as shallow duck holes, the concealment effect may predispose plateau pikas to preferentially utilize longer artificial burrows, although they seldom employ shallow pits for shelter when active burrows are accessible. When used as active burrows, longer burrow tunnels reduce digging costs, potentially leading to slightly higher utilization rates compared to shorter burrows.

Accessibility and ease of excavation may drive plateau pikas' preference for inclined artificial burrows. When threatened by predators, plateau pikas will rapidly retreat into their burrows (Smith and Dobson 2022), making immediate access essential for predator avoidance. Field observations confirm that natural burrow entrances predominantly feature inclined configurations, with vertical entrances being exceptionally rare (Wei et al. 2013). This morphological preference reflects functional advantages, as inclined burrows permit more efficient ingress and egress compared to vertical counterparts. Furthermore, the inclined design facilitates soil removal by plateau pikas, enhancing the usability of these artificial burrows as active burrows.

Translocations have been widely used to establish nonnative species populations and restore native species (Griffith et al. 1989). When introducing plateau pika populations into new areas, we recommend providing both deep and shallow inclined artificial burrows to accommodate the need for both habitat burrows and territorial markings. However, further research is needed to explore how to balance these two requirements.

## 5 Conclusions

Our study demonstrates that rapid vegetation enhancement has no substantial immediate impact on plateau pikas' utilization of artificial burrows, despite vegetation being a critical adaptive factor for burrowing mammals in general. Instead, burrow accessibility and scent‐marking potential—driven by morphological preferences—are the primary determinants shaping their spatial resource selection. These findings suggest that established pika populations may maintain stable underground spatial resource use when facing rapid above‐ground vegetation changes, highlighting a decoupling between rapid surface vegetation dynamics and underground spatial resource utilization in this species. For conservation management, artificial burrows should be designed with optimized morphological features to align with pika's morphological preferences, rather than prioritizing adjustments based solely on vegetation.

## Author Contributions

**Rui Zhang:** conceptualization (equal), data curation (lead), formal analysis (equal), funding acquisition (lead), investigation (lead), methodology (equal), project administration (equal), resources (equal), software (lead), supervision (equal), validation (supporting), visualization (lead), writing – original draft (lead), writing – review and editing (equal). **Wei Liu:** conceptualization (equal), data curation (supporting), formal analysis (equal), funding acquisition (supporting), investigation (supporting), methodology (equal), project administration (equal), resources (supporting), software (supporting), supervision (equal), validation (lead), visualization (supporting), writing – original draft (supporting), writing – review and editing (equal).

## Conflicts of Interest

The authors declare no conflicts of interest.

## Data Availability

The datasets generated during and/or analyzed during the current study are available at https://doi.org/10.6084/m9.figshare.28817492.

## References

[ece372526-bib-0001] Byers, J. E. , K. Cuddington , C. G. Jones , et al. 2006. “Using Ecosystem Engineers to Restore Ecological Systems.” Trends in Ecology & Evolution 21, no. 9: 493–500. 16806576 10.1016/j.tree.2006.06.002

[ece372526-bib-0002] Cai, H. , X. Yang , and X. Xu . 2015. “Human‐Induced Grassland Degradation/Restoration in the Central Tibetan Plateau: The Effects of Ecological Protection and Restoration Projects.” Ecological Engineering 83: 112–119.

[ece372526-bib-0003] Chen, X. Y. , J. Li , S. Q. Wang , et al. 2025. “Response of Plant Phylogenetic Structure to Plateau Pika (*Ochotona curzoniae*) Disturbance on Alpine Meadow of Qinghai‐Tibetan Plateau.” Land Degradation and Development 36, no. 1: 218–230.

[ece372526-bib-0004] Cui, H. , Y. Wang , X. Zhou , and W. Li . 2024. “Positive Role of Plateau Pika (*Ochotona coronae*) on Environmental Quality at Low and Moderate Density on the Tibetan Plateau: Evidence From a Meta‐Analysis.” Journal of Environmental Management 361, no. 121: 239. 10.1016/j.jenvman.2024.121239 38815422

[ece372526-bib-0005] Davidson, A. D. , J. K. Detling , and J. H. Brown . 2012. “Ecological Roles and Conservation Challenges of Social, Burrowing, Herbivorous Mammals in the World's Grasslands.” Frontiers in Ecology and the Environment 10, no. 9: 477–486.

[ece372526-bib-0006] Delibes‐Mateos, M. , A. T. Smith , C. N. Slobodchikoff , and J. E. Swenson . 2011. “The Paradox of Keystone Species Persecuted as Pests: A Call for the Conservation of Abundant Small Mammals in Their Native Range.” Biological Conservation 144: 1335–1346.

[ece372526-bib-0007] Dong, L. , X. Cai , R. Gan , et al. 2025. “Seasonal Variations in the Foraging Strategies of Plateau Pikas (*Ochotona curzoniae*).” Animals 15: 902. 40218296 10.3390/ani15070902 PMC11988150

[ece372526-bib-0008] Gao, Q. , Y. Guo , H. Xu , et al. 2016. “Climate Change and Its Impacts on Vegetation Distribution and Net Primary Productivity of the Alpine Ecosystem in the Qinghai‐Tibetan Plateau.” Science of the Total Environment 554–555: 34–41. 10.1016/j.scitotenv.2016.02.131 26950617

[ece372526-bib-0009] Gao, Q.‐Z. , Y.‐F. Wan , H.‐M. Xu , Y. Li , W.‐Z. Jiangcun , and A. Borjigidai . 2010. “Alpine Grassland Degradation Index and Its Response to Recent Climate Variability in Northern Tibet, China.” Quaternary International 226: 143–150.

[ece372526-bib-0010] George, T. L. , and S. Zack . 2001. “Spatial and Temporal Considerations in Restoring Habitat for Wildlife.” Restoration Ecology 9, no. 3: 272–279.

[ece372526-bib-0011] Griffith, B. , J. M. Scott , J. W. Carpenter , and C. Reed . 1989. “Translocation as a Species Conservation Tool: Status and Strategy.” Science 245, no. 4917: 477–480. 17750257 10.1126/science.245.4917.477

[ece372526-bib-0012] Ivanter, E. V. 2023. “Study of the Water Vole (*Arvicola amphibius*) at the Northwestern Boundary of Its Range.” Biological Bulletin 50, no. 2: 162–174.

[ece372526-bib-0013] Lai, C. H. , and A. T. Smith . 2003. “Keystone Status of Plateau Pikas (*Ochotona curzoniae*): Effect of Control on Biodiversity of Native Birds.” Biodiversity and Conservation 12: 1901–1912.

[ece372526-bib-0014] Li, J. , B. Zhu , L. Ren , Y. Zhang , and Q. Wang . 2025. “A Non‐Destructive Method for Three‐Dimensional Characterizing Plateau Pika's Burrow System.” Journal of Environmental Management 380, no. 124: 789. 10.1016/j.jenvman.2025.124789 40073470

[ece372526-bib-0015] Li, W. , J. M. H. Knops , X. Zhou , et al. 2023. “Anchoring Grassland Sustainability With a Nature‐Based Small Burrowing Mammal Control Strategy.” Journal of Animal Ecology 92: 1345–1356. 37211647 10.1111/1365-2656.13938

[ece372526-bib-0016] Li, X. L. , J. Gao , G. Brierley , Y. M. Qiao , J. Zhang , and Y. W. Yang . 2011. “Rangeland Degradation on the Qinghai‐Tibet Plateau: Implications for Rehabilitation.” Land Degradation and Development 24: 72–80.

[ece372526-bib-0017] McCullough Hennessy, S. , D. H. Deutschman , D. M. Shier , et al. 2016. “Experimental Habitat Restoration for Conserved Species Using Ecosystem Engineers and Vegetation Management.” Animal Conservation 19, no. 6: 506–514.

[ece372526-bib-0018] Menzel, S. 2018. “Artificial Burrow Use by Burrowing Owls in Northern California.” Journal of Raptor Research 52, no. 2: 167–177.

[ece372526-bib-0019] Qin, Y. , B. Huang , W. Zhang , Y. Yu , S. Yi , and Y. Sun . 2021. “Pikas' Burrowing Activity Promotes Vegetation Species Diversity in Alpine Grasslands on the Qinghai‐Tibetan Plateau.” Global Ecology and Conservation 31: e01806.

[ece372526-bib-0020] Qin, Y. , Y. Qin , and S. Yi . 2018. “Application of Ground Penetrating Radar in Studying Plateau Pika Burrows of Typical Alpine Grasslands.” [In Chinese.] Pratacultural Science 35, no. 12: 3013–3019.

[ece372526-bib-0021] Qu, J. , K. Li , M. Yang , W. Li , Z. Yanming , and A. T. Smith . 2007. “Seasonal Dynamic Pattern of Spatial Territory in Social Groups of Plateau Pikas (*Ochotona curzoniae*).” [In Chinese.] Acta Theriologica Sinica 27, no. 3: 215–220.

[ece372526-bib-0022] Smith, A. T. , Badingqiuying , M. C. Wilson , and B. W. Hogan . 2019. “Functional‐Trait Ecology of the Plateau Pika *Ochotona curzoniae* in the Qinghai‐Tibetan Plateau Ecosystem.” Integrative Zoology 14: 87–103. 29316275 10.1111/1749-4877.12300

[ece372526-bib-0023] Smith, A. T. , and F. S. Dobson . 2022. “Social Complexity in Plateau Pikas, *Ochotona curzoniae* .” Animal Behaviour 184: 27–41.

[ece372526-bib-0024] Smith, A. T. , and J. M. Foggin . 1999. “The Plateau Pika (*Ochotona curzoniae*) Is a Keystone Species for Biodiversity on the Tibetan Plateau.” Animal Conservation 2: 235–240.

[ece372526-bib-0025] Smith, A. T. , H. J. Smith , X. Wang , X. Yin , and J. Liang . 1986. “Social Behavior of the Steppe‐Dwelling Blacklipped Pika (*Ochotona curzoniae*).” Acta Theriologica Sinica 6, no. 1: 13–32.

[ece372526-bib-0026] Song, Z. , X. Li , X. Su , and C. Li . 2023. “Analyzing the Recovery Mechanisms of Patchy Degradation and Its Response to Mowing and Plateau Pika Disturbances in Alpine Meadow.” Ecological Indicators 154, no. 110: 565.

[ece372526-bib-0027] Souter, N. J. , C. Michael Bull , and M. N. Hutchinson . 2004. “Adding Burrows to Enhance a Population of the Endangered Pygmy Blue Tongue Lizard, *Tiliqua adelaidensis* .” Biological Conservation 116: 403–408.

[ece372526-bib-0028] Surkova, E. , S. Popov , and A. Tchabovsky . 2019. “Rodent Burrow Network Dynamics Under Human‐Induced Landscape Transformation From Desert to Steppe in Kalmykian Rangelands.” Integrative Zoology 14: 410–420. 30983144 10.1111/1749-4877.12392

[ece372526-bib-0029] Svendsen, G. E. 1976. “Structure and Location of Burrows of Yellow‐Bellied Marmot.” Southwestern Naturalist 20, no. 4: 487–494.

[ece372526-bib-0030] Tang, Z. , Y. Zhang , Z. Zheng , et al. 2024. “Grazing Affects Ecosystem Traits by Regulating Plateau Pika Activities at the Landscape Scale.” Science of the Total Environment 946, no. 174: 356. 10.1016/j.scitotenv.2024.174356 38945235

[ece372526-bib-0031] Wang, X. , and K. Dai . 1990. “A Study on the Breeding Area and the Territorial Behavior in Plateau Pika (*Ochotona curzoniae*).” [In Chinese.] Acta Theriologica Sinica 10, no. 3: 203–209.

[ece372526-bib-0032] Wang, Z. , J. Yan , A. Martin , et al. 2023. “Alpine Grassland Degradation Intensifies the Burrowing Behavior of Small Mammals: Evidence for a Negative Feedback Loop.” Integrative Zoology 19: 240–252. 37243518 10.1111/1749-4877.12730

[ece372526-bib-0033] Wang, Z. , J. Yan , M. Pawley , et al. 2024. “Do Degraded Grasslands Provide a Better Habitat for Plateau Pika? Testing the Nutritional Hypothesis.” Agriculture, Ecosystems and Environment 367, no. 108: 993.

[ece372526-bib-0034] Wei, W. , S. An , Q. Zheng , M. Qin , and T. Chen . 2022. “Structural Changes in Vegetation Coincident With Reseeding *Elymus nutans* Can Increase Perceived Predation Risk of Plateau Pikas (*Ochotona curzoniae*).” Applied Animal Behaviour Science 255, no. 105: 745.

[ece372526-bib-0035] Wei, W. , X. Yao , Y. Zhang , et al. 2023. “Vegetation Restoration Measures: Increasing Plant Height Suppresses Population Densities of Plateau Pikas.” Land Degradation and Development 34: 2201–2213.

[ece372526-bib-0036] Wei, W. , L. Zhang , G. Yang , J. Xu , X. Fan , and W. Zhang . 2013. “A Study on the Burrow Features and Functions of Plateau Pikas.” [In Chinese.] Acta Prataculturae Sinica 22, no. 6: 198–204.

[ece372526-bib-0037] Wei, W. , and W. Zhang . 2018. “Architecture Characteristics of Burrow System of Plateau Pika, *Ochotona curzoniae* .” Pakistan Journal of Zoology 50, no. 1: 311–316.

[ece372526-bib-0038] Wu, P. , H. Zhang , and Y. Wang . 2015. “The Response of Soil Macroinvertebrates to Alpine Meadow Degradation in the Qinghai‐Tibetan Plateau, China.” Applied Soil Ecology 90: 60–67.

[ece372526-bib-0039] Wu, X. , G. Shang , H. Chen , et al. 2023. “Protein Availability on Qinghai‐Tibetan Plateau Meadows Determines Density and Life‐History Characteristics of Plateau Pikas (*Ochotona curzoniae*).” Journal of Mammalogy 104, no. 5: 1112–1123.

[ece372526-bib-0040] Xu, C. , B. R. Silliman , J. Chen , et al. 2023. “Herbivory Limits Success of Vegetation Restoration Globally.” Science 382: 589–594. 37917679 10.1126/science.add2814

[ece372526-bib-0041] Xu, H. , R. Zhang , J. Guo , et al. 2024. “Response Mechanism of Rodent Burrow Density to Natural Environmental Factors in Desert Areas Based on Multisource Data.” Catena 242, no. 108: 91.

[ece372526-bib-0042] Xu, W. , and X. Liu . 2007. “Response of Vegetation in the Qinghai‐Tibet Plateau to Global Warming.” Chinese Geographical Science 17, no. 2: 151–159.

[ece372526-bib-0043] Yao, X. , H. Wang , S. Zhang , M. Oosthuizen , Y. Huang , and W. Wei . 2022. “Impact of Plateau Pika Burrowing Activity on the Grass/Sedge Ratio in Alpine Sedge Meadows in China.” Frontiers in Plant Science 13: 1036438. 36643295 10.3389/fpls.2022.1036438 PMC9838571

[ece372526-bib-0044] Zhang, R. , and W. Liu . 2024a. “How Do Plateau Pikas Use Burrows During Population Reestablishment?” Global Ecology and Conservation 54: e03147.

[ece372526-bib-0045] Zhang, R. , and W. Liu . 2024b. “Preference for Ground Cover When Selecting Burrow Entrances in Plateau Pikas.” Ecology and Evolution 14: e11564. 38895577 10.1002/ece3.11564 PMC11184209

[ece372526-bib-0046] Zhang, R. , W. Liu , and H. Xu . 2018. “Effects of Plateau Pika (*Ochotona curzoniae*) on Alpine Meadow Phytocoenosium and Analysis of the Strategy It Uses to Expand Habitat.” Acta Ecologica Sinica 38: 48–52.

[ece372526-bib-0047] Zhang, R. , H. Xu , and W. Liu . 2018. “Dynamic of the Burrows Distribution During the Restoration of Plateau Pika (*Ochotona curzoniae*) Population.” [In Chinese.] Acta Theriologica Sinica 38, no. 1: 46–55.

[ece372526-bib-0048] Zhou, X. , X. Chen , K. Yang , et al. 2024. “Vegetation Restoration in an Alpine Meadow: Insights From Soil Microbial Communities and Resource Limitation Across Soil Depth.” Journal of Environmental Management 360, no. 121: 129. 10.1016/j.jenvman.2024.121129 38749128

